# Draft genome sequence of *Xylaria bambusicola* isolate GMP-LS, the root and basal stem rot pathogen of sugarcane in Indonesia

**DOI:** 10.1128/mra.01043-23

**Published:** 2024-02-20

**Authors:** Poonguzhali Selvaraj, Vayutha Muralishankar, Sahanna Muruganantham, Jeremy H. F. Tham, Saefudin Saeroji, Endah Susiyanti, Kenny J. X. Lau, Naweed I. Naqvi

**Affiliations:** 1 Temasek Life Sciences Laboratory, and, National University of Singapore, Singapore, Singapore; 2 PT Gunung Madu Plantations, Lampung, Indonesia; University of California Riverside, Riverside, USA

**Keywords:** Xylaria, sugarcane, fungus, pathogenesis, genome analysis, root and basal stem rot

## Abstract

The first draft genome of *X. bambusicola* GMP-LS, the causal pathogen of the Root, and Basal Stem Rot disease in Sugarcane is presented based on single-molecule real-time PacBio sequencing. Xylaria genome (72.43 Mb) is predicted to encode 13,430 proteins and will contribute to molecular understanding of fungal pathogenesis.

## ANNOUNCEMENT

Xylaria (Sordariomycetes, Xylariales, Xylariaceae), the largest cosmopolitan genus of Ascomycota, is defined by teleomorph features such as stromatic perithecia. Xylaria species are mostly found as saprotrophs (plant debris, dung, or termite nests), whereas representation as phytopathogens is uncommon. *X. bambusicola* GMP-LS was isolated from Root and basal stem rot disease (RBSR)-infected sugarcane plantations in Indonesia. An *X. bambusicola* strain was originally described from bamboo in Taiwan (1, 2). We performed the complete *de novo* genome sequencing, assembly, and annotation of this important fungal pathogen of sugarcane.

Fungal isolate GMP-LS was collected and purified from stroma in infected sugarcane in PT Gundung Madu Plantations (Indonesia) and taxonomically identified as *Xylaria bambusicola* based on ITS and LSU rRNA sequence analyses (Fig. S1; DOI 10.5281/zenodo.10317624). High-molecular-weight genomic DNA was extracted from 7-day-old mycelial cultures in Potato dextrose media using MasterPure Yeast DNA extraction kit (Lucigen, Biosearch Technologies, Singapore). The whole-genome DNA library preparation, sequencing, and *de novo* assembly were performed at Macrogen, Singapore. DNA integrity and purity was checked using the picogreen method (Invitrogen) and sequencing quality inspected using FastQC (3). Short reads were generated on the Illumina Novaseq platform (Illumina, USA) using whole-genome shotgun libraries prepared with the Illumina Truseq DNA Nano kit, followed by single-molecule real-time (SMRT) sequencing using the PacBio RS II system.

A total of 9.7 billion bases from 970,008 reads generated from Sequel II platform were submitted for *de novo* assembly that used wtdbg2, v 2.3 (4) and polished using Arrow (5), resulting in 315 contigs with N50 value at 958,750 bp. In addition, a total of 3.42 billion bases from 22.68 million Illumina reads was applied for accurate genome sequence using 3 rounds of polishing in Pilon (v 1.21) (6). The final genome consists of 296 contigs with the length of the longest contig and N50 value being 4,992,453 bp and 959, 283 bp, respectively. The total size of *X. bambusicola* GMP-LS genome was 72,430,123 bp with a mean GC content of 34.9%. Benchmarking Universal Single-Copy Orthologs (BUSCO v 3) (7) homology search against eukaryote_odb10 lineage data set assessed the genome assembly completeness to be 94.90%. Standard default settings were used for the aforementioned software analyses.

Functional annotation using EggNOG-mapper version 2 (8) showed 73.45% hits mainly consisting of carbohydrate, amino acid, nucleotide, and lipid metabolism-related genes. A total of 13,665 genes (Table S1; DOI 10.5281/zenodo.10317624) were predicted of which 98.3% had hits in Uniprot, InterPro, PFAM, or TIGRFAMs database. Average length of proteins was 361 amino acids, with 192 tRNAs and 47 rRNAs predicted in the genome. Notably, it has 44 defense mechanisms and 229 signal transduction-related genes, such as the NLR family and Nacht signaling domain. A total of 400 genes for secondary metabolites synthesis, transport, and metabolism could be predicted such as polyketide synthases that can govern the pathogenicity (Table S2; DOI 10.5281/zenodo.10317624). The availability of this *X. bambusicola* genome will greatly enhance our knowledge of the RBSR disease mechanism and facilitate better understanding of other pathogens within the Xylariales.

## Data Availability

The complete genome sequence and annotation data for Xylaria bambusicola GMP-LS is accessible at DDBJ/EMBL/GenBank under the accession number JAWHQM000000000: https://www.ncbi.nlm.nih.gov/datasets/genome/GCA_033171785.1 and https://ftp.ncbi.nlm.nih.gov/genomes/all/GCA/033/171/785/GCA_033171785.1_ASM3317178v1/ Bioproject number PRJNA1021335, Biosample number SAMN37605086, and its corresponding Sequence Read Archive (SRA) numbers are SRR26289707, SRR26289708, SRR26289709.
