# Erratum for Selvaraj et al., “Draft genome sequence of *Xylaria bambusicola* isolate GMP-LS, the root and basal stem rot pathogen of sugarcane in Indonesia”

**DOI:** 10.1128/mra.00278-24

**Published:** 2024-04-16

**Authors:** Poonguzhali Selvaraj, Vayutha Muralishankar, Sahanna Muruganantham, Jeremy H. F. Tham, Saefudin Saeroji, Endah Susiyanti, Kenny J. X. Lau, Naweed I. Naqvi

## ERRATUM

Volume 13, no. 3, e01043-23, 2024, https://journals.asm.org/doi/10.1128/mra.01043-23. In the second paragraph of the announcement, “PT Gundung Madu Plantations (Indonesia)” should read “PT Gunung Madu Plantations (Indonesia).”

In Acknowledgments, “We thank Sri Haryani and Remaja Sitepu for their invaluable help in this project.” should read “We thank Chong Seng Wai, Teh Choo Pong, and Sri Haryani for their invaluable help in this project.”

